# Human umbilical cord mesenchymal stem cells in diabetes mellitus and its complications: applications and research advances

**DOI:** 10.7150/ijms.87472

**Published:** 2023-09-11

**Authors:** Luyao Li, Jicui Li, Haifei Guan, Hisashi Oishi, Satoru Takahashi, Chuan Zhang

**Affiliations:** 1 Department of Endocrinology, the Second Hospital of Jilin University, Changchun 130041, Jilin, P.R. China.; 2 Department of Nephrology, the Second Hospital of Jilin University, Changchun 130041, Jilin, P.R. China.; 3 Department of Comparative and Experimental Medicine, Nagoya City University Graduate 24 School of Medical Sciences, Aichi 467-8601, Nagoya, Japan.; 4 Institute of Basic Medical Sciences and Laboratory Animal Resource Center, University of Tsukuba, Ibaraki 305-8575, Tsukuba, Japan.

**Keywords:** umbilical cord, mesenchymal stem cells, diabetes mellitus, pluripotent, clinical application, regenerative medicine

## Abstract

Diabetes mellitus and its complications pose a major threat to global health and affect the quality of life and life expectancy of patients. Currently, the application of traditional therapeutic drugs for diabetes mellitus has great limitations and can only temporarily control blood glucose but not fundamentally cure it. Mesenchymal stem cells, as pluripotent stromal cells, have multidirectional differentiation potential, high self-renewal, immune regulation, and low immunogenicity, which provide a new idea and possible development direction for diabetes mellitus treatment. Regenerative medicine with mesenchymal stem cells treatment as the core treatment will become another treatment option for diabetes mellitus after traditional treatment. Recently, human umbilical cord mesenchymal stem cells have been widely used in basic and clinical research on diabetes mellitus and its complications because of their abundance, low ethical controversy, low risk of infection, and high proliferation and differentiation ability. This paper reviews the therapeutic role and mechanism of human umbilical cord mesenchymal stem cells in diabetes mellitus and its complications and highlights the challenges faced by the clinical application of human umbilical cord mesenchymal stem cells to provide a more theoretical basis for the application of human umbilical cord mesenchymal stem cells in diabetes mellitus patients.

## Introduction

Diabetes mellitus (DM) is the most prevalent metabolic disorder caused by the inability of the pancreas to secrete insulin adequately or the body's inability to use insulin effectively. According to the 9th edition of the International Diabetes Federation (IDF) Atlas of Diabetes, approximately 7.002 million adults aged 20-79 years will have DM worldwide by 2045 1. Type 1 diabetes mellitus (TIDM) and type 2 diabetes mellitus (T2DM) are the two most common types of DM. Patients with T1DM are primarily treated with insulin replacement therapy to alleviate absolute insulin deficiency, but may be at risk for hypoglycaemia and tumourigenesis. Human islet transplantation is an effective treatment for T1DM, with a combination of impaired hypoglycaemic awareness and severe hypoglycaemic episodes 2. However, islet transplantation may be greatly limited in clinical application due to a shortage of donor islets and immune rejection. Drug therapy is an important treatment modality for patients with T2DM 3, but its side effects (such as diarrhoea, nausea, vomiting, and anaemia) and drug prices remain to be investigated. Meanwhile, persistent hyperglycaemia can cause chronic damage or dysfunction of the eyes, kidneys, heart, blood vessels, and nerves, and intervention in DM and its complications and reduction of mortality are imminent 4, 5.

Mesenchymal stem cells (MSCs) are widely used in various cell therapies because of their many advantages, such as self-renewal capacity, multispectral differentiation ability, tissue damage repair, and lack of co-stimulatory molecules 6. The abundant source of human umbilical cord mesenchymal stem cells (HUC-MSCs), low ethical controversy, low infection risk, high proliferation and differentiation ability, and very low immunogenicity make them uniquely advantageous for DM therapy. Recently, studies related to the treatment of DM with HUC-MSCs have rapidly developed. This review describes the advantages and mechanisms of HUC-MSCs in treating DM and the application and research progress of HUC-MSCs in DM-related complications, providing more options for managing DM and its complications.

## Source of HUC-MSCs

Umbilical cord blood (UCB) is a valuable stem cell source. MSCs can be isolated from neonatal umbilical cords by enzymatic digestion and show a positive expression of classical MSC surface markers. The umbilical cord comprises the umbilical artery, umbilical vein, Wharton's jelly (WJ), and external amniotic epithelium surrounding the mucus connective tissue 7. MSCs can be isolated from different umbilical cord parts, including the blood, sub-umbilical vein endothelium, and the WJ. Researchers have successfully isolated and cultured MSCs from the perivascular layer of Wharton collagenous vessels of the human umbilical vein 8. MSCs can also be isolated from non-perivascular areas (sub-amniotic membranes) 9. Platelet-derived growth factor (PDGF) produced by human amniotic cells may induce cell migration from the vascular system to the amnion 10. The human umbilical cord is a rich MSC source.

## Advantages of HUC-MSCs

HUC-MSCs have compelling advantages in treating DM, including 1 abundant sources, easy collection, and easy preservation and transportation 11; 2 easy isolation, high purity, and non-tumorigenic 12; 3 high amplification potential 13; 4 functional stability after lyophilisation and recovery 14; 5 no adverse effects of collection on the donor, and ethical issues are circumvented 15; and 6 low probability of infection and transmission of pathogenic microorganisms. In contrast, bone marrow-derived MSCs (BM-MSCs) have a high risk of viral infection and a significant decrease in cell number and proliferation/differentiation capacity with age 16, 17. 7 More primitive and proliferative differentiation capacity. Compared with BM-MSCs, HUC-MSCs have higher pancreatic differentiation potential and proliferative capacity 18. Compared with dental pulp-derived mesenchymal stem cells (PU-MSCs) and adipose tissue-derived mesenchymal stem cells (AD-MSCs), HUC-MSCs have the strongest efficacy in ameliorating glucose and lipid metabolism disorders in T2DM 19. 8 Very low immunogenicity 20. In summary, HUC-MSCs are an ideal source of cells for cell therapy in DM.

## Possible mechanisms of HUC-MSCs for DM treatment

**Figure 1:**
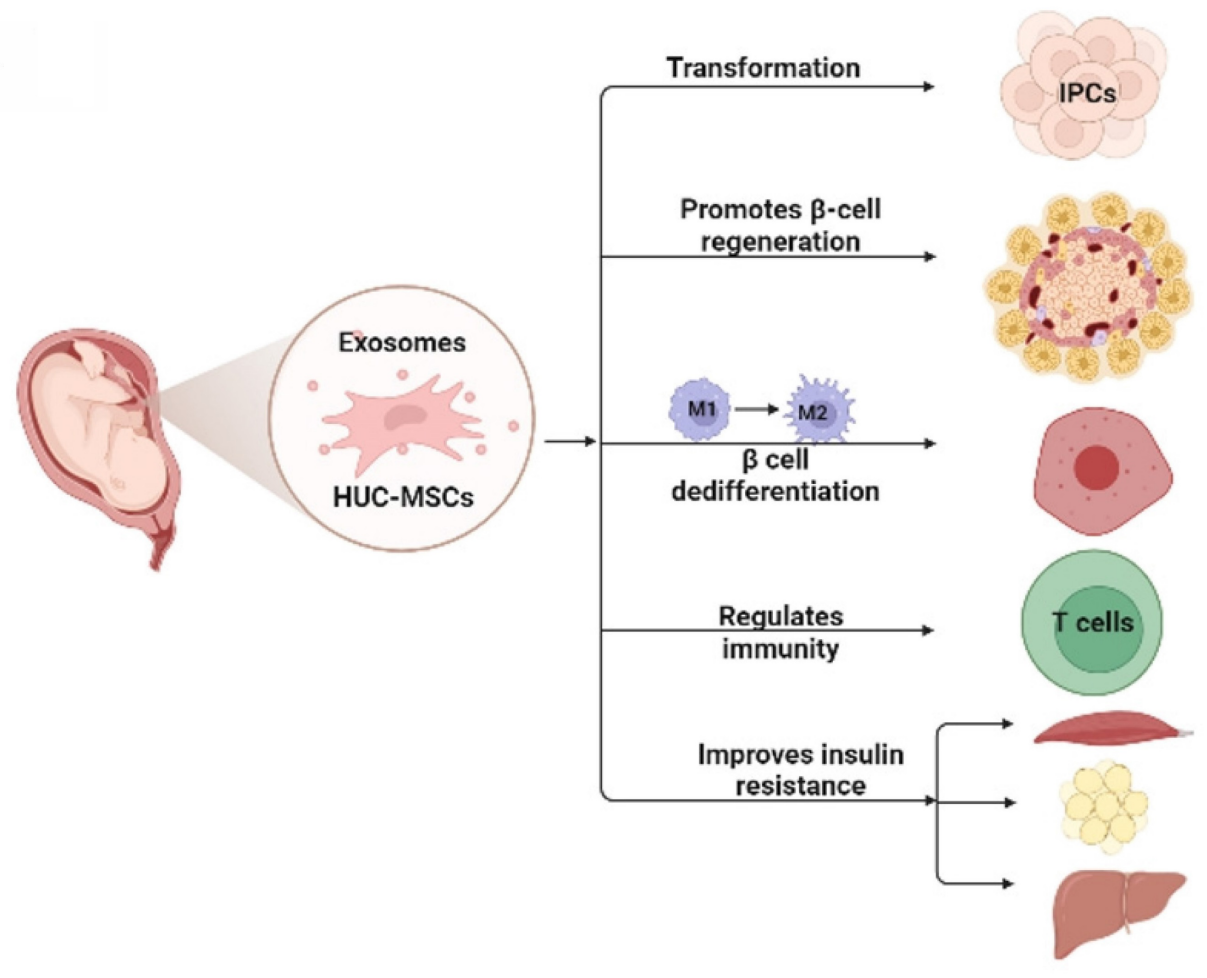
This figure illustrates the possible mechanism of HUC-MSCs for the treatment of DM. HUC-MSCs exert beneficial effects on diabetes by differentiating into IPCs, promoting islet β-cell regeneration, reversing β-cell dedifferentiation, reducing islet β-cell inflammation, and improving insulin resistance. IPCs insulin-producing cells.

MSCs for DM are cell-based therapeutic approaches that have shown remarkable therapeutic effects in DM because of their self-renewal, differentiation potential, and immunosuppressive properties. Numerous studies have shown that HUC-MSCs are a novel strategy to treat DM, and their possible mechanisms 21 include: 1) homing to the damaged pancreas and acting through local nutrition and secretion of paracrine factors; 2) differentiation into insulin-producing cells (IPCs); 3) reversal of beta-cell (β-cell) dedifferentiation, thereby alleviating β-cell dysfunction and protecting islet β-cells; 4) promotion of islet β-cell regeneration; 5) secretion of anti-inflammatory cytokines and macrophage phenotype regulation, thereby reducing islet β-cell inflammation; and 6) enhancing insulin sensitivity in target tissues and improving insulin resistance (Fig. **1**).

### Homing effect of HUC-MSCs

One advantage of MSCs for DM mitigation is their ability to home to damaged tissues and then directly proliferate and differentiate to replace damaged cells and repair damaged tissues. Homing is potentially important for recruiting MSCs to the injury and regeneration sites 22. MSCs homing includes both non-systematic and systemic homing. In non-systematic homing, MSCs are locally transplanted into the target tissue and then directed to the injury site via a chemokine gradient. In systemic homing, the molecular mechanisms of MSCs homing include initial tethering by selectins, activation by cytokines, blockade by integrins, exudation or migration using matrix remodelling agents, and extravasation toward chemokine gradients 23. In 2017, HUC-MSCs labelled with 1,1'-dioctadecyl-3,3,3',3'-tetramethylindocarbocyanine perchlorate (DiI) were detected in the pancreas of T1DM mice, suggesting that HUC-MSCs may target and migrate to damaged organs to exert therapeutic effects 24. Yin et al. pre-labelled HUC-MSCs with cell membrane-Dil (CM-Dil) to demonstrate their migration in various tissues, thus confirming the implantation of MSCs in the pancreatic islets of T2DM mice. This suggests that homing of HUC-MSCs may be closely related to tissue damage 25. Overall, HUC-MSCs may play a role in treating DM by homing to damaged islets. However, the homing rate of MSCs is low, and MSCs may exert protective effects through other mechanisms.

### Paracrine effects of HUC-MSCs

The paracrine properties of MSCs make them a key tissue repair option, and the paracrine effect of MSCs is achieved through the secretion of soluble factors and release of extracellular vehicles (EVs), such as exosomes and microvesicles 26. All the factors secreted by MSCs are called the secretome and comprise various cytokines, chemokines, angiogenic factors, and growth factors. Moreover, up to 80% of the therapeutic effects of MSCs are mediated by paracrine signalling 27. HUC-MSCs secrete soluble molecules such as keratinocyte growth factor (KGF), hepatocyte growth factor (HGF), vascular endothelial growth factor (VEGF), fibroblast growth factor (FGF), placental growth factor (PGF), monocyte chemoattractant protein 1 (MCP-1), insulin-like growth factor 1 (IGF-1), epidermal growth factor (EGF), prostaglandin E2 (PGE2), indoleamine 2,3-deoxygenase (IDO), interleukin-10 (IL-10), interleukin-6 (IL-6), transforming growth factor-β1 (TGF-β1), nitric oxide (NO), human leukocyte antigen-G5 (HLA-G5), tumour necrosis factor-α stimulated gene 6 (TSG-6), and neurotrophic factors 12, 28-30. These factors play a role in promoting tissue regeneration, participating in angiogenesis, promoting ulcer tissue healing, wound healing, modulating immunity, anti-inflammation, anti-apoptosis, and cytoprotection. Recently, there has been intense interest in the synthesis and release of EVs by MSCs via paracrine secretion. Human umbilical cord mesenchymal stem cell-derived exosomes (HucMSC-exs) are nanometer-sized and are capable of rapid diffusion across biological barriers and cell membranes. Numerous studies have shown that HucMSC-exs have anti-inflammatory, anti-apoptotic, tissue repair, neuroprotective, and immunomodulatory properties, suggesting that HucMSC-ex may be a potential DM therapy. HucMSC-ex alleviates T2DM by activating the regenerative capacity of islets 31, improving insulin sensitivity 32, reversing peripheral insulin resistance, and attenuating β-cell destruction 33. Human umbilical cord mesenchymal stem cell-derived small extracellular vesicles (HUC-MSC-sEVs) attenuated structural damage in the pancreas, kidney, and liver of T2DM rats 34. HucMSC-ex protects β-cells from hypoxia-induced apoptosis by carrying miR-21 to attenuate endoplasmic reticulum (ER) stress and inhibit p38 MAPK phosphorylation 35. Exosome-loaded immunomodulatory biomaterials can attenuate the local immune response induced by grafts in DM mice 36. The above studies revealed the potential value of HucMSC-ex and miRs in DM. Overall, HUC-MSCs play a protective role against DM by secreting soluble factors and EVs.

### Differentiation of HUC-MSCs into IPCs

HUC-MSCs ameliorate hyperglycaemia and weight loss in DM rats by differentiating them into IPCs 37-39. HUC-MSCs induced to differentiate into IPCs 40, 41 express pancreatic β-cell differentiation-related genes (e.g. nestin, pancreatic duodenal homeobox-1 (PDX-1), neurogenin3 (NGN3), paired box 6 (PAX6), paired box 4 (PAX4), nk2 homeobox 2 (NKX2.2), nk6 homeobox 1 (NKX6.1), glucose transporter 2 (GLUT-2), and insulin (INS) genes) 42, 43, and promote the secretion of serum C-peptide and INS in DM rats 44, 45. Additionally, HUC-MSCs promote the survival, function, and number of islet-like cell clusters 46. Co-culture of HUC-MSCs with T1DM rat pancreatic cells promotes survival, proliferation, and induced differentiation of HUC-MSCs into IPCs 47. Furthermore, the differentiation of IPCs is a very complex process, and the initial stage of nestin preselection, appropriate induction reagents 48, and extracellular matrix 49 are necessary for the *in vitro* culture of IPCs from HUC-MSCs. PDX-1 50, 51, inhibition of Notch signalling 52 and laminin 411 53 effectively regulate the differentiation of MSCs into IPCs. Under hypoxic conditions, UCB-MSCs also efficiently differentiate into IPCs 54, 55. Additionally, factors that effectively promote the efficacy of IPC action include Port-A catheter transplantation 56, suspension culture 57, and addition of the histone deacetylase (HDAC) inhibitor TMP269 58. Overall, HUC-MSCs can replace damaged islet β cells by inducing differentiation into IPCs, which are ideal seed cells to treat DM.

### HUC-MSCs can effectively improve islet β-cell function

β-Cell dedifferentiation is thought to be an important contributor to β-cell dysfunction in T2DM 59. Pro-inflammatory cytokines can lead to β-cell dysfunction and de-differentiation. MSCs reduce endogenous interleukin-1b (IL-1b) production in T2DM islets by secreting IL-1Ra, thereby reducing islet injury and reversing β-cell dedifferentiation 60. Additionally, it has been shown that the interleukin-1 receptor antagonist (IL-1Ra) can also regulate the phenotypic transition of macrophages 61. In db/db mice, early infusion of HUC-MSCs reduced β-cell dedifferentiation markers, such as aldehyde dehydrogenase 1 family member A3+ (ALDH1A3+), and increased the proportion of Ins+ β cells and Pdx1+/Ins+ cells 62. This suggests that MSCs transplantation may be a therapeutic strategy for protecting and restoring β-cell function in patients with T2DM. Additionally, the potential mechanisms for the therapeutic effects of MSCs on DM may involve islet regeneration, including direct differentiation into functionally competent β-cells. Pax4, in concert with Pdx1, Ngn3, and MAF bZIP transcription factor A (MafA), can induce the differentiation of HUC-MSCs into pancreatic β-like cells (pβLCs) functional pancreatic β cells 63. MSCs participate in the repair process by secreting various cytokines and growth factors with paracrine and autocrine activities, which may contribute to endogenous β-cell regeneration and islet structural recovery 21. Wei et al. found that HUC-MSCs protect islets from hypoxia-induced dysfunction 64 and secrete IGF-1 to exert a trophic effect on islets 65. Bao et al. found that HUC-MSCs overexpressing tissue inhibitors of matrix metalloproteinase (TIMP)-1 induced weight loss and hypoglycaemia and improved islet function and survival in T1DM mice 66. Lu et al. found that HUC-MSC transplantation is safe and effective in T1DM patients and may better protect residual β-cells 67. Hu et al. found that the combination of HUC-MSCs and selegiline was effective in improving hyperglycaemia, promoting islet β-cell regeneration, and inhibiting islet alpha cell (α-cell) production in T2DM rats 68. Although the exact mechanism needs to be further explored, this study may provide a new therapeutic approach for DM.

### Interaction of HUC-MSCs with various immune cells and cytokines

#### HUC-MSCs and macrophage polarization

DM is characterised by mild chronic inflammation, which is often accompanied by inflammatory cell infiltration in islets. Macrophage infiltration of islets and autoimmune destruction of β-cells are important features of the chronic inflammatory process in T1DM. Macrophages may be a major contributor to the development of chronic inflammation and insulin resistance in patients with T2DM 3. UC-MSC transplantation induces an increase in M2 macrophages in pancreatic islets, adipose tissue, liver, and skeletal muscle. HUC-MSCs produce anti-inflammatory mediators and growth factors that suppress inflammation and improve insulin sensitivity and β-cell regeneration 25. HUC-MSCs reduce insulin resistance by secreting IL-6 69 and IL-10 70 to promote M2 macrophage polarization. MCP-1 secreted by HUC-MSCs synergistically regulates macrophage polarisation with IL-6 71. Additionally, low-dose decitabine may prolong the antidiabetic effects of MSCs and promote sustainable β-cell recovery by polarising macrophages to the M2 phenotype 72. Overall, HUC-MSCs can reduce islet β-cell inflammation by polarising macrophages to the M2 anti-inflammatory phenotype, thereby alleviating islet dysfunction in patients with DM.

#### HUC-MSCs and other immune cells

MSCs not only act on innate immune cells but also interact with other immune cells, thus regulating multiple effector functions 73. MSCs regulate antigen presentation by dendritic cells (DCs), cytotoxicity of natural killer (NK) cells and neutrophil activation. MSCs induce peripheral tolerance in T cells and exert effective tissue protection through the release of anti-inflammatory, anti-apoptotic, and trophic molecules 74. Li et al. found that regulatory T cells (Treg)/T helper cell 17 (Th17) and Treg/T helper cell 1 (Th1) cell ratios increased significantly after 4 weeks of transplantation of HUC-MSCs, while the Th17/Th1 cell ratio remained unchanged 75, suggesting that HUC-MSCs ameliorate immune disorders in T2DM by repairing Treg cells. HUC-MSCs can reduce blood glucose, increase C-peptide levels, and Treg production in T2DM patients 76, suggesting that HUC-MSCs with powerful immunomodulatory ability are safe and effective in T2DM patients, and microencapsulated HUC-MSCs reduce effector Th1 cells and repair the Treg/Th17 ratio 77, suggesting that HUC-MSCs may treat T1DM by modulating immunity. In addition, HUC-MSCs have shown efficacy in other autoimmune diseases, such as T1DM combined with Sjogren syndrome (SS) 78, 79. Overall, MSCs may represent a new strategy to treat immune-mediated diseases.

### HUC-MSCs improve insulin resistance

Insulin resistance (IR) is one of the most common and important pathological features of T2DM. MSCs can exert immunomodulatory and anti-inflammatory effects through paracrine effects, thereby increasing insulin sensitivity and improving insulin resistance in T2DM rats 80. Umbilical cord mesenchymal stem cell-conditioned medium (UC-MSC-CM) may improve IR in C2C12 cells by improving glucose transporter 4 (GLUT4) translocation, insulin signalling pathways, and mitochondrial content and function 81. HUC-MSCs also improve IR by modulating the balance between PTEN-mediated PI3K/Akt and ERK/MAPK signalling pathways 82. UC-MSCs infusion and fasting-mimicking diet (FMD) synergistically modulate the systemic inflammatory microenvironment and improve hyperglycaemia and lipid metabolism disorders in T2DM mice 83. Glucagon-like peptide-1 (GLP-1) gene modification of HUC-MSCs improves fasting glucose, IR, and β-cell function in T2DM mice 84. HUC-MSCs combined with liraglutide can downregulate the TLR4/NF-kB inflammatory pathway and oxidative stress while improving glucose metabolism and inhibiting islet β-cell apoptosis in an ASK1/JNK/BAX pathway-dependent manner in T2DM rats 85, 86. In conclusion, HUC-MSCs act as an effective treatment for T2DM by improving IR, thereby providing a potential avenue for developing novel clinical T2DM therapies.

## HUC-MSCs and other types of diabetes

Recently, with advances in regenerative medicine research, HUC-MSCs may provide a new treatment option for other types of DM. Hu et al. found that HUC-MSCs therapy could restore the function of residual islet β cells in patients with new-onset T1DM over a longer period. This suggests that implantation of HUC-MSCs is expected to be an effective strategy for treating new-onset T1DM 87. Yang et al. found that HUC-MSCs reduced inflammatory responses and attenuated pancreatic injury in rats with severe acute pancreatitis (SAP) 88. Kong et al. found that HUC-MSCs ameliorated chronic pancreatitis in rats via the AKT-mTOR-S6K1 signalling pathway, which provides a basis for the clinical application of HUC-MSCs in treating pancreatitis 89. In 2019, HucMSC-ex delivered exogenous miR-145-5p to inhibit pancreatic ductal adenocarcinoma progression, suggesting a therapeutic role of HUC-MSCs in pancreatic exocrine diseases 90. Additionally, transplantation of HUC-MSCs can effectively alleviate weight loss symptoms, reduce blood glucose levels, and improve offspring survival in gestational diabetes mellitus (GDM) patients 91. However, it has been shown that GDM adversely affects the proliferative capacity and viability of HUC-MSCs 92. Therefore, to address this situation, it is crucial to identify conditions that improve the survival of HUC-MSCs, reduce apoptosis, and promote proliferation.

In conclusion, MSC therapy presents a novel approach for treating DM, displaying substantial efficacy in both basic and clinical trials. HUC-MSCs exhibit the capacity to migrate towards damaged pancreatic islets, facilitated by homing and paracrine effects, thus assuming a reparative role. Additionally, they induce differentiation into IPCs, replacing impaired islet β-cells and enabling the secretion of C peptide and insulin. Furthermore, they counteract β-cell de-differentiation, thereby safeguarding pancreatic β-cells, and facilitate regeneration of islet β-cells along with structural revitalization, consequently enhancing islet β-cell functionality. Their impact extends to immune cells, encompassing macrophages, DCs, NK cells, neutrophils, and T cells, thereby exerting immunomodulatory and anti-inflammatory properties. This therapeutic modality also ameliorates insulin resistance by targeting insulin-responsive organs. In summary, the collective mechanisms through which HUC-MSCs operate synergistically culminate in an amelioration of diabetic symptoms (Table. **1**). Nevertheless, the homing rate of MSCs remains limited, prompting the need for further investigations to enhance their homing rate, bolster their survival rate post-transplantation, and optimize overall efficacy and safety. Notably, contemporary research endeavors have augmented the efficacy of diabetes mellitus treatment via preemptive treatments of MSCs, including hypoxic pre-conditioning. While current studies yield promising clinical outcomes, the full spectrum of optimal efficacy warrants deeper exploration.

## HUMSCs and complications of T2DM

### Diabetic nephropathy

Diabetic nephropathy (DN) is one of the most serious complications of DM and a major cause of end-stage chronic kidney disease. HUC-MSCs act mainly by promoting paracrine mechanisms, such as mitogenic, anti-fibrotic, anti-inflammatory, antioxidant, anti-apoptotic, cytoprotective and immunomodulatory. HUC-MSC transplantation is expected to be an effective therapeutic approach for preventing and treating DN. An et al. found that HUC-MSCs lowered blood glucose, improved renal function and renal histopathological changes in DN nonhuman primates 93. Fang et al. found that IGF-1 secreted by HUC-MSCs promoted renal tubular cell proliferation and reduced apoptosis, thus exerting a protective effect on the kidneys 94. Additionally, HUC-MSCs inhibited the levels of inflammatory factors IL-6, IL-1β, tumour necrosis factor-alpha (TNF-α), TGF-β, MCP-1, and nuclear factor-κB (NF-κB) and downregulated the expression of fibronectin alpha-smooth muscle actin (α-SMA) and collagen IV, suggesting that HUC-MSCs benefit podocytes under high glucose (HG) by suppressing inflammation and fibrosis while delaying the progression of DN 95-97. Nie et al. found that HUC-MSCs decreased malondialdehyde levels and 4-hydroxynonenal (4-HNE) protein expression and increased the antioxidant enzymes catalase (CAT) and glutathione peroxidase (GPX) 98. HUC-MSCs attenuated the expression of TGF-β1, α-SMA, collagen I, and heat shock protein 47 (HSP47) mRNA and increased the expression of E-cadherin and bone morphogenetic protein 7 (BMP-7) mRNA, suggesting that HUC-MSCs can prevent renal injury in DN rats via paracrine humoral factors 99, 100. Notably, HUC-MSCs improved renal function in mice, mainly due to immunomodulatory effects rather than direct implantation and trans-differentiation into renal cells 101. Overall, HUC-MSCs can improve DN through the above-mentioned mechanisms, and may be a promising DN therapeutic strategy.

### Diabetic retinopathy

Diabetic retinopathy (DR) is a common cause of visual impairment and blindness in working-age individuals. Microangiopathy and inflammatory responses are key components of DR. Recently, MSCs have received increasing attention for their tissue damage repair therapy, anti-inflammatory effects, and pro-angiogenic effects, and they offer potential options for the treatment of DR. HUC-MSCs play an anti-inflammatory role and inhibit retinal neuronal apoptosis by upregulating the expression of adiponectin (APN) and neurotrophin-4 (NT-4) and downregulating the expression of myocardial infarction-associated transcript (MIAT), IL-1β, IL-6, and high-sensitivity C-reactive protein (hs-CRP) 102, 103. HUC-MSCs increase the number of surviving retinal ganglion cells (RGCs) and improve neuroprotection through a BDNF-dependent mechanism, suggesting that HUC-MSCs may slow DR progression through paracrine humoral factors 104, 105. Additionally, numerous studies have shown that HucMSC-ex have anti-inflammatory, anti-apoptotic, tissue repair, neuroprotective, and immunomodulatory properties. Moreover, EVs are nanometer-sized and can diffuse rapidly through the retina 106. Fu et al. found that HucMSC-ex effectively prevented early retinal vascular damage and retinal thickening, and alleviated DM-induced structural damage to the retina 107. Li et al. found that HucMSC-derived exosomes shuffled microRNA-17-3p ameliorated the inflammatory response and oxidative damage in DR mice by targeting STAT1, providing new insights into novel targeted therapies for DR 108. In 2021, HucMSC-derived exosomes shuffled microRNA-18b exerted anti-apoptotic and anti-inflammatory effects in DR rats by mediating the MAP3K1/NF-κB axis, suggesting that miR-18b is critical for HucMSC-ex treatment of DR 109. Zhang et al. found that HucMSC-ex overexpressing miR-126 was able to reduce hyperglycemia-induced retinal inflammation by targeting and regulating high mobility group box 1 (HMGB1) 110. Overall, these studies have laid a solid foundation for HUC-MSCs in DR treatment.

### Diabetic central nervous system complications

DM is a risk factor for acute stroke and can lead to a higher risk of ischaemic stroke and a worse prognosis 111-113. Cerebral haemorrhage, neurological deficits, and white matter (WM) damage can be severe after stroke in DM mice 114. Inflammatory and immune responses play important roles in ischaemic stroke prognosis, and human umbilical cord blood cells (HUCBCs) are widely accepted to repair the central nervous system 115. Stem cell-rich HUCBCs can survive, migrate, differentiate, and restore neurological function in the ischaemic brain microenvironment of stroke rats 116. Lin et al. found that CD34-immunosorted human umbilical cord blood haematopoietic stem cells (HUCB34) after hypoxic preconditioning promoted neuronal progenitor cell (NPCs) homing to the ischaemic brain and enhanced neuronal synapse regeneration 117. Chen et al. found that HUCBCs promote vascular and WM remodelling by upregulating miR-126 expression while promoting M2 macrophage polarisation and inducing neural repair by decreasing vascular cell adhesion molecule-1 (VCAM-1) and MCP-1 expression 118. HUCBCs increase the density of oligodendrocyte progenitors and oligodendrocytes, increase angiopoietin 1 (Ang-1), expression, and decrease the expression of ischaemic border zone (IBZ) RAGE, matrix metalloproteinase 9 (MMP-9), and toll-like receptor 4 (TLR4), suggesting that HUCBCs have a therapeutic effect on nerve repair in DM rats with stroke 119, 120. Therefore, MSCs therapy may be a promising therapeutic option for diabetic patients with central nervous system complications.

### Diabetic autonomic neuropathy

Diabetic autonomic neuropathy (DAN) is a serious and common complication of DM that has significant adverse effects on patient survival and quality of life 121. Diabetic cystopathy (DC) is considered a manifestation of diabetic neuropathy, and its pathogenesis may be related to long-term hyperglycaemia, bladder wall remodelling induced polyuria, and oxidative stress leading to smooth muscle cell and neuronal damage 122. Wu et al. found that HUC-MSCs overexpressing nerve growth factor (NGF) could secrete neurotrophic factors and cytokines in the rat spinal cord and could also differentiate into NeuN neurones and glial fibrillary acidic protein (GFAP)-positive astrocytes to effectively prevent bladder hypertrophy and remodelling, thereby reversing the progression of DC and restoring bladder function 123. Shin et al. found that HUC-MSC transplantation improved urinary function in DM rats, which provides a rationale for HUC-MSC treatment of DM-related detrusor underactivity (DUA) 124. Wu et al. found that HUC-MSC transplantation may improve diabetic erectile dysfunction in rats by increasing the production of paracrine growth factors (VEGF), endothelial nitric oxide synthase (eNOS), IGF1, and basic fibroblast growth factor (bFGF) 125. In conclusion, transplantation of HUC-MSCs may be a new potential therapeutic option for DAN.

### Diabetic foot disease

Diabetic foot ulcers (DFU) are full-length lesions that occur in the skin of the foot of patients with DM, accompanied by infection and tissue destruction caused by neuropathy and/or peripheral artery disease (PAD) 126. DFU has a high disability and mortality rate, which severely affects the quality of life of patients, shortens life expectancy, and imposes a heavy socioeconomic burden 127, 128. In recent years, HUC-MSCs have achieved good therapeutic effects in the treatment of DFU. Zhao et al. found that HUC-MSCs specifically homed to ulcerated tissue and promoted epithelialisation of ulcerated tissue, possibly by stimulating the release of cytokeratin 19 from keratin-forming cells and promoting extracellular matrix formation 129. Shi et al. found that HUC-MSCs promoted wound healing in DFU rats by transdifferentiating, regulating inflammation, and providing growth factors that promote angiogenesis, cell proliferation, and collagen deposition 130. Xia et al. found that HUC-MSCs prevented or cured foot ulcers in DFU rats by reversing the neuronal structure and function by upregulating NGF and promoting significant angiogenesis in the femoral nerve-innervated gastrocnemius muscle 131. HUC-MSCs induce angiogenesis 132, 133, promote tissue repair and regeneration 134, and reduce muscle damage and apoptosis in the ischaemic hind limbs of DM mice 135. Transplantation of HUC-MSCs significantly improved skin temperature, ankle-arm pressure index, transcutaneous partial pressure of oxygen, and claudication distance in patients with postoperative diabetic foot disease. This is accompanied by a significant increase in neovascularization and complete or gradual ulcer healing 136. In conclusion, transplantation of HUC-MSCs may be a potential strategy for clinical application in DFU, although its long-term effects remain to be elucidated.

### Impaired wound healing in DM

Impaired wound healing is a common DM complication. DM is associated with persistent inflammation and a defective tissue repair response. Impaired angiogenesis is an important factor in delaying chronic diabetic wound healing. Poorly healing wounds in DM mice exhibit a persistent inflammatory response, a deficiency of M2 macrophages 137, 138, a prolonged accumulation of pro-inflammatory M1 macrophages, elevated levels of pro-inflammatory cytokines and proteases, and reduced levels of various growth factors 139-141. HUC-MSCs can self-renew, multi-directionally differentiate, and secrete multiple cytokines and growth factors, and their mechanisms to improve diabetic wound healing mainly include 1) promoting diabetic wound healing by differentiating into keratin-forming cells 142; 2) secreting molecules related to wound healing in a paracrine manner (VEGF, PDGF, KGF, TGF-β1, SMA, scavenger receptor class B type1 (SR-B1), and platelet endothelial cell adhesion molecule-1 (PECAM-1/CD31)) to promote angiogenesis 143-146; 3) regulating the activity, function, and proliferative capacity of vascular endothelial cells by reducing oxidative stress and inflammatory response, thereby promoting angiogenesis 147, 148; 4) inducing functional recovery of vascular endothelial cells by modulating macrophage phenotype 149; and 5) stimulating diabetic fibroblast activity and promoting cell proliferation, collagen synthesis, and glycosaminoglycan levels, thereby playing a role in skin wound healing play a role 150. Moreover, HUC-MSCs may be more effective than fibroblasts in stimulating diabetic wound healing 151, 152. Additionally, HUC-MSCs accelerate wound healing in diabetic rats by increasing epidermal and dermal thickness and density, accelerating epithelial and collagen regeneration, and increasing angiogenesis 153. Han et al. found that the Wnt signalling pathway activation promoted the proliferation and differentiation of HUC-MSCs, thereby facilitating the healing of diabetic skin wounds 154. Yue et al. found that c-Jun overexpression promotes the proliferation and migration of HUC-MSCs *in vitro* and accelerates diabetic wound closure, re-epithelialization, and angiogenesis in vivo 155. The development of new technologies has extensively improved the therapeutic efficacy of HUC-MSCs. HUC-MSCs can improve skin wound healing in diabetic mice by combining with Pluronic F127 hydrogel 156, tissue-engineered scaffolds 157, 158, or Cas9-AAV6 engineering modification 159. Overall, HUC-MSC transplantation may have a therapeutic effect on impaired diabetic wound healing; however, its specific therapeutic modalities and safety need to be further explored.

## HUC-MSCs infusion is safe and effective for COVID-19 with diabetes

Currently, coronavirus disease 2019 (COVID-19) is a serious global public health problem and is significantly associated with an increased risk of developing DM 160. At the same time, patients with DM are at a high risk of developing severe COVID-19 infections, have a complex disease process, and have significantly higher mortality rates 161, 162. Severe COVID-19 is thought to result from the hyper-inflammatory state and overactive immune response caused by severe acute respiratory syndrome coronavirus 2 (SARS-CoV-2) infection, as well as cytokine storm and immune thrombosis. The SARS-CoV-2 spike glycoprotein binds to angiotensin-converting enzyme 2 (ACE2), and the serine protease transmembrane protease serine 2 (TMPRSS2) initiates S proteins that can facilitate viral entry into cells, viral replication, and cell-to-cell transmission 163. The activity of ACE2 is increased in DM mice 164, 165 and significantly increased in patients with DM treated with angiotensin-converting enzyme inhibitors (ACEI) and angiotensin receptor blockers (ARB) 166, suggesting that patients with DM may be at increased COVID-19 risk.

MSCs can achieve immunomodulation by secreting various cytokines through paracrine pathways or by interacting directly with the immune cells 167. ACE2 and TMPRSS2 were expressed at low levels in HUC-MSCs, suggesting that HUC-MSCs may have the ability to "evade" viral infection and thus exert immunomodulatory effects 168. HUC-MSCs reduced the levels of inflammatory molecules associated with the COVID-19 "cytokine storm", including interferon-γ (IFNγ), IL1β, IL-6, and TNFα, and regulated upon activation of normal T cell expressed and secreted factor (RANTES). Additionally, no serious adverse events related to HUC-MSC infusion have been observed 169, 170. HUC-MSCs improved respiratory distress and reduced inflammatory biomarkers in patients with critically ill COVID-19-induced automated resources directory service (ARDS) 171. Tao et al. found that UC-MSCs significantly increased pulmonary static compliance, maintained a stable partial pressure of oxygen (PaO2)/fraction of inspired oxygen (FiO2) ratio, and improved renal function in critically ill COVID-19 patients, suggesting that UC-MSCs transplantation may have a positive therapeutic effect in critically ill COVID-19 patients 172. In conclusion, HUC-MSC therapy may be a potential treatment option for DM combined with COVID-19.

## Opportunities and challenges

HUC-MSCs have shown impressive results in treating DM and its complications, but most studies are still in the preclinical stage. Improving the survival and efficacy of HUC-MSCs after transplantation in a challenging metabolic environment may be an interesting topic in the future, as elevated palmitate levels in the sera of obese and T2DM patients lead to a shift from an immunosuppressive to an immunostimulatory state in MSCs, suggesting that the metabolic disease environment alters the immunomodulatory efficacy of healthy donor MSCs 173. Boland et al. found that culturing HUC-MSCs in xeno-free conditions attenuated palmitate-induced impairment of the immunomodulatory function of HUC-MSCs 174. Additionally, the mode of MSCs administration affects therapeutic efficacy, with intravenous delivery methods being more effective than intraperitoneal grafts 175. Local delivery causes MSCs to cluster into "spheroids", thereby altering gene expression and phenotype. In 2020, researchers found that budesonide could act synergistically with prostaglandin E2 (PGE2) produced by spheroid MSCs to inhibit T cell proliferation at the PGE2 receptors EP2 and EP4 176. Moreover, IPCs may be immunogenic and trigger immune responses after transplantation into the host owing to changes in the immune microenvironment and immune cell infiltration, thus reducing cell survival and further differentiation 177. However, encapsulation of IPCs with alginate has been shown to avoid graft rejection, which greatly improves the efficacy of allogeneic or xenogeneic MSCs in the treatment of DM 178.

Stem cell banking is the most important life resource for human beings, which can provide high-quality seed cell resources for stem cell therapy. By establishing a standardized production process of MSCs, it can improve stem cell preparation quality and promote the sustainable development of stem cell clinical applications. Actively promoting the clinical translation of stem cell therapy and improving the survival rate and efficacy of HUC-MSCs after transplantation will become the top priority of stem cell technology research nowadays. With the development of technology, the field of stem cell research has become a frontier hotspot. The implantation of the bioartificial pancreas 179, the labelling of nanoparticles (NP) 180, and the co-microencapsulation of HUC-MSCs/human pancreatic islet-derived progenitor cells (hIDC) 181 may provide new tools for cellular therapy of DM. Carboxylic acid-functionalized single-walled carbon nanotubes (f-SWCNT-COOH) 182 and some cytokines 183 can increase the viability and *ex vivo* expansion of hematopoietic stem cell (HSC) and/or hematopoietic stem progenitor cell (HSPC). These studies provide new perspectives for developing DM cell transplantation therapies based on HUC-MSCs. We believe that the effectiveness of HUC-MSC therapy will be greatly improved by applying advanced technologies such as gene modification, nanotechnology, magnetic targeting technology, and tissue engineering technology. We believe that soon, HUC-MSCs may provide a better solution for the clinical treatment of DM and its complications, and thus can bring new hope to a greater extent in DM patients worldwide.

## Conclusions

**Figure 2:**
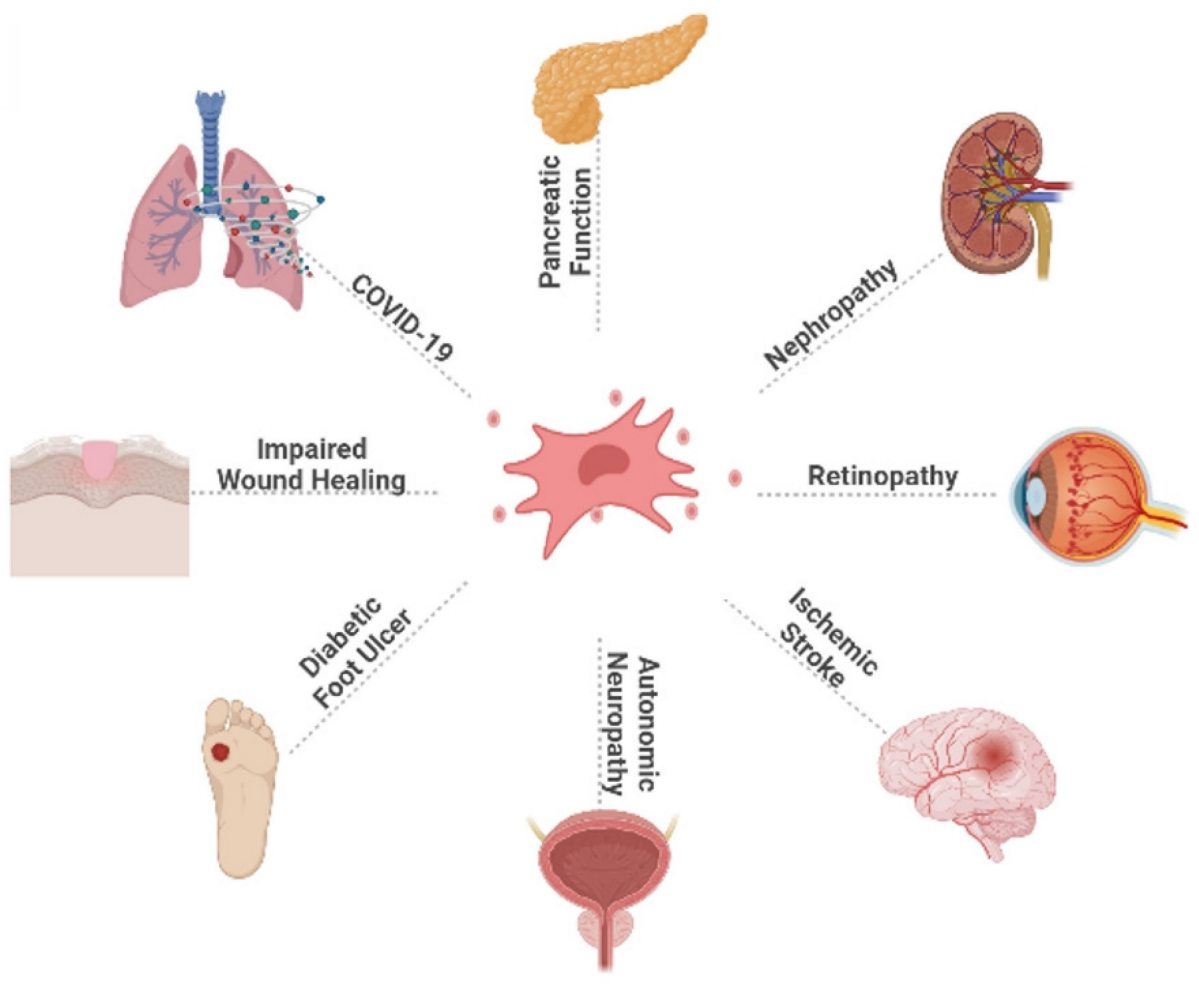
This figure illustrates the broad effect of HUC-MSCs on DM and its complications, as well as the therapeutic effect of HUC-MSCs on diabetic patients infected with COVID-19. COVID-19: coronavirus disease 2019.

The DM epidemic and its complications pose a major threat to global health, accompanied by high morbidity and mortality. Currently, there are many ways to treat DM, such as traditional oral hypoglycaemic therapy and insulin injections, but they can only temporarily control blood glucose levels and cannot cure diabetes, and have insufficient control over diabetic complications, in addition to long-term use of hypoglycaemic drugs or insulin injections, which significantly reduces patient compliance. Recently, regenerative medicine with MSCs treatment as the core has provided new ideas and possible development directions for DM treatment. The characteristics of HUC-MSCs, such as abundant source, less ethical controversy, lower risk of infection, higher proliferation and differentiation ability, and very low immunogenicity, make them stand out among MSCs of different tissue sources. We mainly describe the application of HUC-MSCs in DM and its complications. HUC-MSCs transplantation is expected to be an efficient and ideal treatment for DM and its complications, and its application area will gradually expand (Fig. **2**). Currently, HUC-MSCs therapy is still in the exploration stage, and further research is needed to improve the homing rate, survival rate, efficacy, and safety of MSCs after transplantation. With the gradual maturation of technology and theory, the fundamental treatment of diabetes will usher in a greater breakthrough. We believe that HUC-MSCs transplantation can provide more options for the management of DM and its complications and bring longer-term benefits to patients.

## Figures and Tables

**Table 1:** The possible modes of action of MSCs in the treatment of diabetes are discussed in the table.

Mode of action of HUC-MSCs			
Mode of action		Mechanism	References
**Homing effects**	Systemic homing	Initial tethering by selectins Activation by cytokines Blockade by integrins Exudation or migration using matrix remodelling agents Extravasation toward chemokine gradients	(22, 23)
	Non-systematic homing	Directed to the injury site via a chemokine gradient.	
**Paracrine effects**	Secrete soluble molecules (KGF, HGF, VEGF, FGF, PGF, MCP-1, IGF-1, EGF, PGE2, IDO, IL-10, IL-6, TGF-β1, NO, HLA-G5, TSG-6, and neurotrophic factors)	Promoting tissue regeneration and angiogenesis Promoting ulcer tissue healing and wound healing Modulating immunity, anti-inflammation, anti-apoptosis, and cytoprotection.	(12, 28-30)
	Release of EVs, such as exosomes and microvesicles	Activating the regenerative capacity of islets Improving insulin sensitivity Reversing peripheral insulin resistance Attenuating β-cell destruction	(31-33)
**Differentiation into IPCs**	Induced to differentiate into IPCs	Replace some damaged islet β-cells to secrete C peptide and INS	(40, 45)
**Improve islet β-cell function**	Protection of pancreatic islet beta cells	Secreting IL-1Ra to reduce islet injury and reverse β-cell dedifferentiation	(60, 62)
	Promoting the regeneration of pancreatic islet beta cells	Induced to differentiate into pβLCs functional pancreatic β cells	(63)
**Immunomodulatory effects**	Macrophage	Suppress inflammation and improve insulin sensitivity by secreting IL-6 and IL-10 to promote M2 macrophage polarization.	(25, 69-71)
	DCs, NK cells, neutrophil, and T cells	Regulate antigen presentation by DCs Regulate cytotoxicity of NK Regulate neutrophil activation Induce peripheral tolerance in T cells	(74)
**Improve insulin resistance**	Liver, fat and skeletal muscle	Improve IR in C2C12 cells and improve lipid metabolism disorders in T2DM mice	(81, 83)

HUC-MSCs, Human umbilical cord mesenchymal stem cells; KGF, Keratinocyte growth factor; HGF, Hepatocyte growth factor; VEGF, Vascular endothelial growth factor; FGF, Fibroblast growth factor; PGF, Placental growth factor; MCP-1, Monocyte chemoattractant protein 1; IGF-1, Insulin-like growth factor 1; EGF, Epidermal growth factor; PGE2, Prostaglandin E2; IDO, Indoleamine2,3-deoxygenase; IL-10, Interleukin-10; IL-6, Interleukin-6; TGF-β1, Transforming growth factor-β1; NO, Nitric oxide; HLA-G5, Human leukocyte antigen-G5; TSG-6, Tumor necrosis factor α stimulated gene 6; EVs, Extracellular vehicles; β-cell, Beta cell; IPCs, Insulin-producing cells; INS, Insulin; IL-1Ra, Interleukin-1 Receptor antagonist; PβLCs, Pancreatic β-like cells; DCs, Dendritic cells; NK cells, Natural killer cell; IR, Insulin resistance; T2DM, Type 2 diabetes mellitus.

## Abbreviations

DM: diabetes mellitus
IDF: international diabetes federation
T1DM: type 1 diabetes mellitus
T2DM: type 2 diabetes mellitus
MSCs: mesenchymal stem cells
HUC-MSCs: human umbilical cord mesenchymal stem cells
UCB: umbilical cord blood
WJ: wharton's jelly
PDGF: platelet derived growth factor
BM-MSCs: bone marrow-derived mesenchymal stem cells
PU-MSCs: pulp-derived mesenchymal stem cells
AD-MSCs: adipose tissue-derived mesenchymal stem cells
β-cell: beta cell
DiI: 1,1'-dioctadecyl-3,3,3',3'-tetramethylindocarbocyanine perchlorate
CM-Dil: cell membrane-dil
EVs: extracellular vesicles
KGF: keratinocyte growth factor
HGF: hepatocyte growth factor
VEGF: vascular endothelial growth factor
FGF: fibroblast growth factor
PGF: placental growth factor
MCP-1: monocyte chemoattractant protein 1
IGF-1: insulin-like growth factor 1
EGF: epidermal growth factor
PGE2: prostaglandin E2
IDO: indoleamine2,3-deoxygenase
IL-10: interleukin-10
IL-6: interleukin-6
TGF-β1: transforming growth factor-β1
NO: nitric oxide
HLA-G5: human leukocyte antigen-G5
TSG-6: tumor necrosis factor α stimulated gene 6
HucMSC-ex: human umbilical cord mesenchymal stem cell-derived exosome
HUC-MSCs-sEVs: human umbilical cord mesenchymal stem cell-derived small extracellular vesicle
ER: endoplasmic reticulum
PDX-1: pancreatic duodenal homeobox-1
NGN3: neurogenin3
PAX6: paired box 6
PAX4: paired box 4
NKX2.2: nk2 homeobox 2
NKX6.1: nk6 homeobox 1
GLUT-2: glucose transporter 2
INS: insulin
HDAC: histone deacetylase
IL-1b: interleukin-1b
IL-1Ra: interleukin-1 receptor antagonist
MafA: MAF bZIP transcription factor A
PβLCs: pancreatic β-like cells
TIMP: tissue inhibitors of matrix metalloproteinase
α-cell: alpha cell
DCs: dendritic cells
NK: cytotoxicity of natural killer cell
Treg: regulatory T cells
Th17: t helper cell 17
Th1: t helper cell 1
SS: sjogren syndrome
UC-MSC-CM: umbilical cord-mesenchymal stem cell-conditioned medium
GLUT4: glucose transporter 4
FMD: fasting-mimicking diet
GLP-1: glucagon-like peptide-1
SAP: severe acute pancreatitis
GDM: gestational diabetes mellitus
DN: diabetic nephropathy
TNF-α: tumor necrosis factorTh17- alpha
NF-κB: nuclear factor-κB
α-SMA: alpha-smooth muscle actin
HG: high glucose
HNE: 4-hydroxynonenal
CAT: catalase
GPX: glutathione peroxidase
HSP47: heat shock protein 47
BMP-7: bone morphogenetic protein 7
DR: diabetic retinopathy
APN: adiponectin
NT-4: neurotrophin-4
MIAT: myocardial infarction-associated transcript
hs-CRP: high-sensitivity C-Reactive Protein
RGCs: retinal ganglion cells
HMGB1: high mobility group box 1
WM: white matter
HUCBCs: human umbilical cord blood cells
HUCB34: CD34-immunosorted human umbilical cord blood hematopoietic stem cells
NPCs: neuronal progenitor cells
VCAM-1: vascular cell adhesion molecule-1
Ang-1: angiopoietin 1
IBZ: ischemic border zone
MMP-9: matrix metalloproteinase 9
TLR4: toll-like receptor 4
DAN: diabetic autonomic neuropathy
DC: diabetic cystopathy
NGF: nerve growth factor
GFAP: glial fibrillary acidic protein
DUA: detrusor underactivity
eNOS: endothelial nitric oxide synthase
bFGF: basic fibroblast growth factor
DFU: diabetic foot ulcer
PAD: peripheral artery disease
SR-B1: scavenger receptor class B type1
PECAM-1/CD31: platelet endothelial cell adhesion molecule-1
SARS-CoV-2: severe acute respiratory syndrome coronavirus 2
ACE2: angiotensin converting enzyme 2
TMPRSS2: transmembrane protease, serine 2
ACEI: angiotensin converting enzyme inhibitor
ARB: angiotensin receptor blocker
IL-2R: interleukin-2R
IL-8: interleukin-8
IFNg: interferon-g
IFNγ: interferon-γ
RANTES: regulated upon activation normal T cell expressed and secreted factor
ARDS: automated resources directory service
PaO2: partial pressure of oxygen
FiO2: fraction of inspiration oxygen
PGE2: prostaglandin E2
NP: nanoparticles
hIDC: human pancreatic islet-derived progenitor cells
f-SWCNT-COOH: carboxylic acid functionalized single walled carbon nanotubes
HSC: hematopoietic stem cell
HSPC: hematopoietic stem progenitor cell
